# One Year of Peer Support Work in Forensic Mental Health – Evaluation of Implementation

**DOI:** 10.1192/j.eurpsy.2022.886

**Published:** 2022-09-01

**Authors:** P. Walde, C. Benz, J. Hadala, B. Völlm

**Affiliations:** Universitätsmedizin Rostock, Klinik Für Forensische Psychiatrie, Rostock, Germany

**Keywords:** Peer Support Work, Compulsory Treatment, Addicted Offenders, Forensic Mental Health

## Abstract

**Introduction:**

Peer Support Work can be an effective way to support patients and their participation also in psychiatric populations. Unlike in general psychiatry there is less experience with peer support work in forensic mental health inpatient settings. Characteristics different from general psychiatry, e.g., regarding safety, might be a reason for the delay of their implementation.

**Objectives:**

We aim to present the implementation of a peer support worker in a forensic mental health setting for addicted offenders. We address reservations of staff before the implementation and their development during the first year. The perspective of patients about their experiences is taken into consideration. The development of the peer support workers position and tasks is demonstrated.

**Methods:**

Focus groups and interviews were conducted with several groups of people, amongst them employees of several professions, patients and the peer support worker of the clinic. Interviews and focus groups were recorded and transcribed for thematic analysis.

**Results:**

Reservations of staff comparable to these found in general psychiatry occurred in the forensic mental health professionals. These could be diminished during the first year. Most of the patients were able to accept and trust the peer support worker, in some cases after initial mistrust. The peer support worker felt accepted in the team and was able to develop a routine as well as own tasks.

**Conclusions:**

The experiences from one year testified that implementation of peer support work into a forensic mental health inpatient setting is possible. Further patient outcomes are to be explored but the current results are promising.

**Disclosure:**

No significant relationships.

